# A Deep Brain Stimulation Trial Period for Treating Chronic Pain

**DOI:** 10.3390/jcm9103155

**Published:** 2020-09-29

**Authors:** Prasad Shirvalkar, Kristin K. Sellers, Ashlyn Schmitgen, Jordan Prosky, Isabella Joseph, Philip A. Starr, Edward F. Chang

**Affiliations:** 1 Department of Anesthesiology (Pain Management), University of California San Francisco, San Francisco, CA 94143, USA; Jordan.Prosky@ucsf.edu; 2 Department of Neurological Surgery, University of California San Francisco, San Francisco, CA 94143, USA; Kristin.Sellers@ucsf.edu (K.K.S.); Ashlyn.Schmitgen@ucsf.edu (A.S.); Isabella.Joseph@ucsf.edu (I.J.); Philip.Starr@ucsf.edu (P.A.S.); Edward.Chang@ucsf.edu (E.F.C.); 3 Department of Neurology, University of California San Francisco, San Francisco, CA 94143, USA

**Keywords:** deep brain stimulation, chronic pain, lead externalization, trial period, stereoEEG

## Abstract

Early studies of deep brain stimulation (DBS) for various neurological disorders involved a temporary trial period where implanted electrodes were externalized, in which the electrical contacts exiting the patient’s brain are connected to external stimulation equipment, so that stimulation efficacy could be determined before permanent implant. As the optimal brain target sites for various diseases (i.e., Parkinson’s disease, essential tremor) became better established, such trial periods have fallen out of favor. However, deep brain stimulation trial periods are experiencing a modern resurgence for at least two reasons: (1) studies of newer indications such as depression or chronic pain aim to identify new targets and (2) a growing interest in adaptive DBS tools necessitates neurophysiological recordings, which are often done in the peri-surgical period. In this review, we consider the possible approaches, benefits, and risks of such inpatient trial periods with a specific focus on developing new DBS therapies for chronic pain.

## 1. Introduction

Deep brain stimulation (DBS), which involves the insertion of electrical leads into important cortical or subcortical structures to treat various neurological diseases, is often performed in a single surgery where the leads are subsequently connected to an implanted stimulator. Alternatively, DBS can be performed in a two-staged surgery where the leads (or electrodes) are first implanted, externalized (i.e., the non-neural side of the electrode exits the body), and connected to an external stimulator for a trial period to assess therapeutic benefit over 3–10 days. DBS trials with lead externalization have been used extensively over the last 50 years specifically for treating chronic pain [1,2,3]. Lead externalization permits inpatient testing of stimulation effects prior to permanent implantation, which is especially important for novel or unapproved DBS indications to ensure successful therapeutic response. This is especially important for diseases that encompass broad domains of symptoms spanning from neuropsychiatric to somatic, such as chronic pain syndromes. Because optimal brain targets for chronic pain are still unknown, such trials offer a key opportunity for the exploration and validation of both targets and pulse parameters that may modulate activity in multiple, relevant brain networks underlying various symptoms. Further, such a trial period allows the recording of neurophysiological activity from critical brain regions to further research and understand the mechanisms of action of stimulation. Modern recordings have aimed to uncover key biomarkers of chronic pain states toward the development of adaptive (or “closed-loop”) control algorithms where stimulation delivery is adjusted in response to biomarkers to increase efficacy or avert the development of long-term tolerance.

Such trial periods that allow patients to “test drive” the neuromodulation therapy are used even when the general target regions are known, such as in spinal cord stimulation (SCS) [4]. A trial period is often necessary because of the large heterogeneity in patient response, to assess safety, and for the fine tuning of electrode position relative to the neural target so that patient benefit can be maximized before committing to an expensive therapy. It is worth pointing out that a short trial period to determine chronic brain stimulation targets assumes that an acute response to stimulation can be found (within minutes or hours of stimulation) and that these effects will translate to long-term efficacy. It remains to be determined whether this is the case for chronic pain.

The global burden of chronic pain is significant and growing alongside that of non-communicable diseases, which account for 78.6% of years lived with disability worldwide [5]. The economic impact of chronic neurological conditions such as chronic pain and epilepsy is greater than that of many other health conditions due to absenteeism, reduced levels of productivity, and increased risk of leaving labor markets, indicating that there is a significant indirect economic cost associated with these conditions [6]. Chronic pain also poses a significant cost to the healthcare system; analysis of a Canadian database indicates that the costs attributed to the healthcare of patients with painful neuropathic disorders were considerably higher than those in patients without chronic pain in the same age and sex demographics [6]. Perhaps most importantly, chronic neurological conditions have a significant debilitating effect on the overall quality of life of patients and are associated with some of the poorest quality-of-life indices, with the potential to impact social relationships, economic participation, and mental health. DBS for pain is still performed with a very limited trial period (off-label in the U.S.) resulting in highly variable success rates across patients.

In this review, we outline important considerations for DBS trial periods for chronic pain including possible brain targets for stimulating and recording. We then propose an argument for the use of stereoelectroencephalography (sEEG) in the trial and discuss possible trial related risks. An sEEG based approach is elaborated in the context of characterizing pain biomarkers and neurophysiological effects of stimulation. Finally, we offer practical advice for conducting a trial, including mitigating the placebo effect and rigorously ensuring therapeutic efficacy before permanent implantation.

## 2. Anatomical Targets/Considerations

Determining the optimal neural targets for stimulation-induced pain relief is a key goal of the DBS trial period. The trial period affords the opportunity to explore multiple pain-relevant brain regions based on pre-clinical and human experience to select the sites most appropriate for the pain syndrome and patient. In a survey of the current state of DBS for chronic pain, Farrell et al. focus on three prominent brain targets that have been tested most frequently: the ventral posterolateral/medial thalamus (VPL/VPM), the periventricular/periaqueductal grey (PVG/PAG), and the anterior cingulate cortex (ACC) across a wide variety of chronic pain syndromes [7]. Most studies highlighted in that review used a simple readout of somatic pain intensity (numeric or visual analog rating scale (NRS, VAS)); however, this approach fails to measure changes in the affective or cognitive domains of pain. Numerous other targets have been proposed such as motor cortex, ventral striatum, and insula. We will briefly discuss the most promising candidates below in the context of how these targets may offer pain relief across multiple dimensions of the pain experience. Importantly, the target efficacy for pain relief depends on the class of pain syndrome being treated and the unique qualities of the patient.

The motivation for treating intractable pain with brain surgery began with early results [8] demonstrating analgesia after thalamotomy and selective ablation. Following development by Mazars [9], Hosobuchi et al. used thalamic stimulation to produce successful masking of facial pain with electrically induced paresthesia in four of five patients, with pain relieving effects observed after a few minutes of stimulation [10]. Similar paresthesia-based analgesia for phantom limb pain has been reported previously [11]. Whereas VPL/VPM stimulation induces a phenomenon of purportedly “pleasant” paresthesia, PVG/PAG has been shown to induce a sense of warmth in painful areas. The pain relief following PAG/PVG stimulation has been attributed to the release of endogenous opioids as evidenced by abolished effect after naloxone [12]. In contrast, a study of 45 patients receiving chronic PAG or PVG stimulation for a wide variety of pain syndromes for one year demonstrated that despite the development of long-term tolerance to stimulation in twelve patients, there was no cross-tolerance with morphine [13]. This suggests that pain relief with PAG/PVG stimulation may not exclusively depend on an endogenous opioid mechanisms. Regardless, two large clinical trials evaluating combined VPL/PAG stimulation for chronic pain of various syndromes failed to meet therapeutic endpoints, though these studies were fraught with design and follow-up problems (see [14] for a discussion). Specifically, these trials targeted the same two brain regions across hundreds of patients with vastly different pain syndromes without placebo or randomized design. Notably, a significant portion of patients were lost to follow-up; the resulting patient attrition severely reduced power to detect a clinically meaningful effect and resulted in no significant benefit using an intention-to-treat analysis. These flaws highlight the need for individualized brain targeting based on etiology and the use of endpoints that measure multiple dimensions of pain.

Studies from Aziz and colleagues have ignited interest in targeting the affective dimension of pain by targeting the dorsal ACC. Patients suffering from a variety of chronic neuropathic pain syndromes were followed for up to 3 years, and significant benefit was found at the 6-month time point [2], though no significant effect was found on further follow-up. Further ACC stimulation at the wide pulse widths and high amplitudes required for analgesia was associated with de novo epilepsy in a handful of patients [15]. However, pain relief with ACC stimulation has been reported to have a long wash-in period (hours to 3 days), which may limit the ability to detect a beneficial effect in shorter timescales (however, see [16]). Nonetheless, the novelty of the ACC as a target has inspired a renewed interest in modulating the affective dimension of pain and basic research into pain-relevant mechanisms in the ACC. It is still unknown whether targeting other regions of the ACC may be beneficial for chronic pain despite functional imaging evidence that more anterior or posterior regions may be involved [16,17]; such alternative regions of the cingulate merit exploration for therapeutic DBS in a potential trial period.

Motor cortex stimulation (MCS) has also been studied for pain relief using both non-invasive [18] and direct cortical stimulation [19]. In a meta-analysis, 54% of 117 patients with central pain and 68% of 44 patients with trigeminal neuropathic pain experienced a greater than 40% reduction in pain scores, suggesting efficacy for neuropathic pain syndromes [20]. In contrast, double-blinded studies of MCS failed to show significant analgesia for the treatment of hemi-body post-stroke pain or post-herpetic neuralgia pain, while noting that patients suffering from facial pain and upper limb pain may obtain relief (e.g., see [21]). Potential mechanisms of analgesia resulting from MCS are still debated. Other targets such as the posterior insula have demonstrated increased thermal pain thresholds in epilepsy patients without chronic pain, though generalization to clinical pain states is unknown [22].

When selecting neural targets for deep brain stimulation trial periods, it is important to consider the specific type of chronic pain that the patient is suffering from. Certain types of pain such as facial and upper limb neuropathic pain may warrant targeting the contralateral motor cortex, while inflammatory pain syndromes may benefit relatively more from targeting the PAG. Stimulation of the ACC or insula are other potentially promising targets, though no systematic study has been able to demonstrate long-term pain relief through DBS, suggesting neural adaptation as a key obstacle. The only double-blind sham-controlled clinical trial for post-stroke pain targeted the ventral striatum/capsular region and aimed to mitigate the affective component of pain to improve pain-related disability [23]. At present, while there are numerous individual anecdotes of patients obtaining pain relief from the stimulation of various targets, there is a lack of clear evidence pointing to reliable targets for long-term pain relief. Advances in longitudinally stable pain biomarker detection will ideally inform next-generation closed-loop DBS therapy for chronic pain that may avert long-term tolerance [19,20,24]. The hope is that through individual “N-of-1” DBS trial periods, we may find brain targets that produce acute pain relief that further extends to longer time periods.

## 3. DBS Trial Period Safety and Risks

Trialing deep brain stimulation therapy in advance of a permanent implant can pose additional patient-specific and systemic risks both in relation to the surgery and potential success of this therapy as an option of last resort. Specifically, a DBS trial period may introduce the added risk of possible infection and additional costs of extended inpatient evaluation.

Beyond the risks from intracranial implantation, which can be mitigated with strict planning and surgical protocols, it is important to consider the costs of a temporary implant and inpatient trial period from a holistic perspective. For example, while we are studying the effects of DBS on pain, it is important to control for external factors, such as the effects of financial stress, social isolation, and physical restrictions that are associated with prolonged inpatient stay. However, by accounting for these effects, testing DBS in a temporary, resource-rich environment, and applying the predictive power of an inpatient trial, we may avoid the unnecessary financial and psychosocial burden of a long-term implant for unsuccessful trial patients. Thus, when assessing potential patients for DBS therapy for chronic pain, it is useful to consider the safety, appropriateness, fiscal neutrality, and effectiveness (SAFE) principles for neuromodulation therapies [25].

The SAFE (safety, appropriateness, fiscal neutrality, and effectiveness) principles can be used as an evidence-based algorithm to guide the selection of appropriate pain syndromes and DBS targets for individual patients. These principles further support using a minimally biased trial period to evaluate for benefit, similar to techniques used in SCS for chronic pain treatment. For example, studies using effective psychosocial screening for SCS trials produced a higher rate and longer duration of pain relief than studies in which no psychosocial screening was performed. Further, disregarding these psychosocial factors may increase the risk of injury to patients while creating unnecessary costs from ineffective therapeutic interventions. Thus, performing a well-designed, comprehensive inpatient trial in line with the SAFE principles, as described, can increase the chances of long-term success of DBS for chronic pain. By closely examining each of these guiding principles, as demonstrated below, both patients and researchers can perform a more thorough and personalized approach to trial DBS candidacy.

Safety: Reports of infection rates involved in temporary lead externalization have been mixed. An early single center, prospective analysis over a four-year period found an increased rate of infection when DBS for Parkinson’s disease was performed using a staged procedure with externalization (15.3% vs. 4.2% for one-stage) [26]. However, in a two-stage surgical procedure, Rosa et al. found that lead externalization for 2–7 days did not increase post-operative infection risk following Stage I bilateral DBS lead implant when oral antibiotics were used for 5 days after Stage II lead extender and internal pulse generator (IPG) placement [27]. In this study of 105 patients, the rate of infection from this two-stage surgery (2.8%) was consistent with post-operative risk of infection in the literature. Bojanic et al. similarly observed no increased risk of infection when directly comparing externalized trial vs. one-stage DBS surgery [28]. Of 60 patients undergoing single-stage surgery, they observed 7 infections, while 86 patients undergoing an externalized trial resulted in 3 infections.

Appropriateness and efficacy: A temporary trial stage presents a measurable predictor of long-term clinical benefit. In order to ensure that the trial has realistic predictive value, however, it is critical to ensure a clinically meaningful benefit. Observation of a clinically significant benefit (a 12-point improvement on the 100-point visual analog scale [29]) relies on the consideration of intrinsic factors such as patient attentional biases, motivations, and study expectations. The expectation effects of pain or benefit from DBS can be understood through a motivation–decision model of pain, in which the threat of pain or promise of relief is a consequence of a computed decision to respond to or ignore this pain signal [30]. This is an especially important consideration when working with patients with chronic pain, who have been shown to bias expectations toward pain escape/avoidance and, thus, increase perceived pain intensity through top–down modulatory circuits. In fact, the patient’s expectation of improvement at trial entry may be the single most robust predictor of reported pain reduction. Since anticipation of pain often becomes a self-fulfilling prophecy, addressing patient expectations should be incorporated into the trial plan. The trial stage presents an extended opportunity to use comprehensive psychosocial assessments, including the Structured Clinical Interview for DSM-5 (Diagnostic and Statistical Manual of Mental Disorders—Fifth Edition, SCID-5), Hamilton Depression Rating Scale, West Haven Yale Multidimensional Pain Inventory (WHYPI), Pain Catastrophizing Scale, Beck Depression Inventory, and Beck Anxiety Inventory, in addition to cognitive and behavioral testing to provide a more complete picture of patient outcomes.

Psychosocial components of pain, such as attention, culture, anxiety, and depression, similarly influence the perception of pain and the outcomes of therapies. Despite meeting all clinical criteria, sound surgical approaches, and even early signs of clinical benefit, a significant number of patients continue to fail neuromodulation therapy in the long term. This suggests that the inherent characteristics of patients are a strong factor in determining neuromodulation efficacy. Strict inclusion and exclusion criteria and extensive psychological evaluation are critical prior to trial enrollment [31]. Studies suggest that a comprehensive program consisting of pre-operative psychosocial assessment and consistent psychological and rehabilitative support throughout the trial phase and subsequent therapy are key elements for the success of neurostimulation.

Fiscal neutrality: Bojanic et al. found that despite the extra costs of seven additional inpatient days (up to £11,200), there were significant cost savings when compared to committing patients to a potentially ineffective implant (up to £147,000), particularly when the indication was chronic pain. At our institution, a similar DBS trial period of 7 days presents an actual cost of $34,000 including imaging, surgery, device, and inpatient costs ($150,000 billed to private insurance) versus a cost for a one-stage DBS implant of $90,000 ($203,000 billed to private insurance). With a trial to permanent implant conversion ratio of 50% (higher end), this represents a cost savings of up to $56,000 per every 2 patients ($53,000 when billed to private insurance). The concept of fiscal neutrality (maximizing the neuromodulation approach to be cost neutral in the long term), supports performing adequate trials for DBS for treating pain.

## 4. Motivation for a StereoEEG Trial in Patients with Chronic Pain

sEEG is most commonly used as a diagnostic tool for patients with refractory epilepsy. Localization of seizure onset zones is achieved using multi-contact depth electrodes, which are placed through burr holes targeting both cortical and deep structures of the brain. Continuous recording can provide a three-dimensional view of the origin and spread of epileptic seizures [32]. sEEG has been used to guide treatment of refractory epilepsy when non-invasive treatments such as pharmacology, diet, or other alternative therapeutic options are ineffectual. Similarly, we propose using sEEG in patients with chronic pain who have failed to achieve relief of symptoms with pharmacological interventions or spinal cord stimulators.

sEEG has been widely adopted internationally, with global studies generating extensive data in support of sEEG with potential synergistic applications in cognitive neurophysiology research. sEEG is an invasive monitoring technique, and a major concern is the potential for hemorrhagic complications, which can lead to neurological deficits or death. However, rates of clinically significant hemorrhage and other infections occur at much lower rates in sEEG compared to those in other neurophysiological surgical approaches (e.g., subdural strip and grid electrode implants). sEEG is often a highly efficacious and cost-efficient procedure with a low associated morbidity that has demonstrated success in network characterization for thousands of epilepsy patients worldwide.

**Figure 1:**
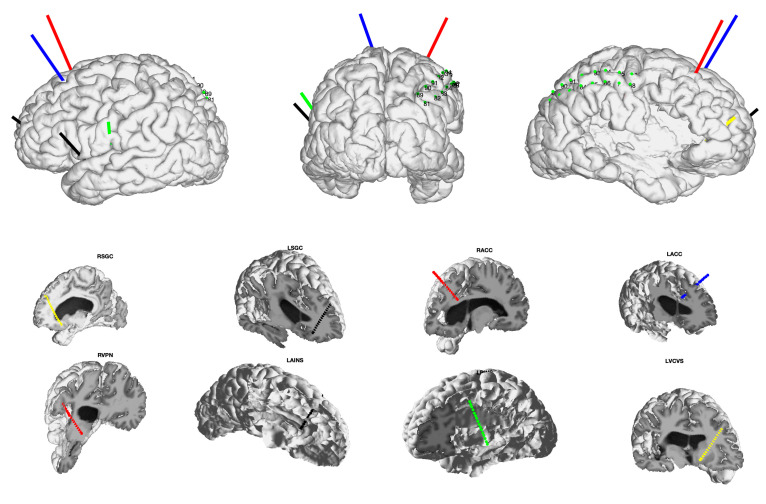
Stereoelectroencephalography (sEEG) targeting for deep brain stimulation (DBS) for chronic pain during the inpatient trial period. Top row depicts left lateral, posterior, and right lateral views in an example patient with post-stroke pain. Eight images across rows 2 and 3 depict individual electrode trajectories targeting labeled regions of interest. R/LSGC: right /left subgenual cingulate cortex, R/LACC: right/left anterior cingulate cortex, RVPN: right ventral posterior thalamic nucleus, LAINS: left anterior insula, LPINS: left posterior insula, LVCVS: left ventral capsule and ventral striatum.

sEEG is an appealing approach for neural circuit mapping and testing acute stimulation efficacy, which we propose can be used to identify optimal brain regions in candidate patients who are most likely to respond when a chronic DBS device is implanted. Specifically, for chronic pain, which engages multiple well-established nodes across a widespread pain network, an sEEG trial can be used to target multiple brain regions based on converging evidence from pre-clinical and human brain mapping studies (Figure 1, see Section 2, Anatomical Targets/Considerations). Similar prior efforts have been successful for epilepsy and even facilitated the development of new responsive brain stimulation therapy [33]. As sEEG is beneficial for intracranial investigations that require sampling from superficial and deep structures simultaneously across both hemispheres, we propose using sEEG as both a clinical tool and to advance research on the fundamental mechanisms of chronic pain processing in patients.

The main benefit of sEEG over other approaches lies in the potential for focal stimulation combined with pain-related network discovery. Methods such as functional magnetic resonance imaging (fMRI) and magnetoencephalography (MEG) can be informative and useful in network discovery, potentially prior to a trial period, to discover optimal targets in each individual patient prior to electrode implantation. Regarding network discovery with other modalities, blood oxygen level dependent (BOLD) signals in fMRI can provide whole-brain localization and temporal tracking of neural activity correlated with pain states [34,35]. However, fMRI-based signals cannot be detected in the ambulatory setting, limiting their use in clinically relevant timescales for chronic pain therapy (however, see [36] for longitudinal fMRI). For example, due to the limitations of patient tolerance and equipment availability, fMRI cannot feasibly be used continuously over a period of hours, making it unrealistic for evaluating clinically relevant spontaneous pain fluctuations. Likewise, prior work has used simultaneous stimulation (with DBS or transcranial magnetic stimulation (TMS)) and recording with magnetoencephalography (MEG) [37] or scalp EEG [38] to identify predictive biomarkers of subjective pain experience without the associated risks of trial surgery. Studies evaluating network effects of DBS with EEG/MEG nonetheless require permanent DBS surgery, which poses greater risks than sEEG. Prior efforts to identify biomarkers of stimulation using TMS/EEG require averaging over larger volumes of tissue and can suffer more from stimulation artifact during simultaneous neural recording. Furthermore, TMS itself can be painful, confounding interpretation of any putative pain-relevant biomarkers. Non-invasive approaches remain an important complement to sEEG and, in theory, could even be used to identify key anatomical pain nodes in the future. For example, studies aiming to decode subjective pain intensity have been able to use MEG successfully to identify local field potential (LFP)-based biomarkers of pain, which are often reproducible within subjects [39]; such individualized MEG mapping may serve a useful pre-surgical step for target planning. While sEEG is limited by the number of electrodes and sampled regions per patient when compared to more global brain network approaches such as fMRI or MEG [40], it can better facilitate the characterization of biomarkers of clinical pain and stimulation with high spatiotemporal resolution.

## 5. Detecting Biomarkers of Chronic Pain and Stimulation-Related Pain Relief

While considerable effort has been devoted to identifying intracranial biomarkers of pain, the vast majority of studies to date have focused on experimental pain states in healthy individuals, which do not necessarily translate to altered brain dynamics seen in patients with chronic pain [34,41]. Rather than focusing exclusively on signatures of evoked pain, we suggest that studying the dynamics of spontaneous pain can be more informative. By further combining simultaneous recording and stimulation, it may be possible to dissociate neural correlates of the pain state from those of stimulation-related pain relief. Such converging evidence would help to develop practical approaches to personalized, adaptive neurostimulation.

### 5.1. Biomarkers of Chronic Pain and Network Discovery

Chronic pain is associated with both spontaneous and evoked pain, which are associated with distinct underlying brain activity. The chronic pain experience is almost certainly a network-wide phenomenon, but focusing on critical nodes of this network has pointed to brain regions that may harbor useful biomarkers [42]. fMRI studies provide evidence that brain activation patterns of sustained chronic pain states do not overlap with those of stimulus-evoked acute pain states in both patients and healthy subjects [43]. Baliki et al. found a double dissociation where sustained chronic low back pain in patients was associated with increased BOLD activity in the medial prefrontal cortex (mPFC) and rostral cingulate, while thermal experimental pain preferentially activated the insula both in patients and healthy controls. Further, the posterior thalamus and bilateral dorsolateral prefrontal cortex (DLPFC) exhibit atrophy over time in chronic pain [44]; pain-related activation of the DLPFC is also related to activity in the mPFC, but not in the insula [43]. The duration of experienced pain also influences brain representation of pain. Compared to patients experiencing subacute low back pain for only 2 months, patients with chronic back pain lasting >10 years show increased brain activity in the mPFC, orbitofrontal cortex, precuneus, and amygdala [45]. These authors and others have argued that the chronification of pain shifts brain representations from nociceptive to emotional circuits. Therefore, long-term chronic pain is associated with network-level changes in pain processing that cannot necessarily be inferred from solely studying evoked pain in healthy patients using experimental stimuli such as laser-evoked potentials or the thermal grill illusion. These data suggest that the sensory, cognitive, and emotional phenomenology of real-world chronic pain is distinct from that of experimentally induced pain. As such, the mPFC, rostral ACC, and DLPFC may be sensible targets to detect biomarkers of the chronic pain state.

Recordings of LFP from brain regions that are likely to harbor chronic-pain-relevant signals (e.g., mPFC, ACC, DLPFC) can be used to further understand mechanisms of pain and devise control strategies for closed-loop algorithms. Two brain stimulation devices with closed-loop functionality are current available: Neuropace^®^ RNS (Neuropace, Mountain View, CA, USA) [46] and Medtronic^®^ Percept (Medtronic, Minneapolis, MN, USA) (recently approved for sensing and stimulation [47] and full closed-loop functionality is anticipated soon). Theses device are capable of using neural power band-limited time series signals (e.g., alpha band power) as inputs to an embedded closed-loop controller that can modify stimulation parameters to optimize therapy. For this reason, we focus on band powers of interest that have previously been associated with predicting pain states.

Despite the limitations of interpreting biomarkers of acute/evoked pain state, there is an abundance of such data using sEEG, MEG, and EEG, which can provide a starting point. The most commonly reported neural features predictive of a high pain state have been a decrease in the central alpha band (8–12 Hz) power [48,49,50] and an increase in the parietal or prefrontal gamma band (>30 Hz) power [51,52]. Importantly, recent studies have distinguished neural correlates of painful stimuli from nonpainful stimuli, providing greater selectivity for pain states. Using sEEG, Liu et al. found significant evoked-pain-related cross-frequency coupling between theta (4–8 Hz) and gamma band activity in the amygdala and hippocampus, which can be tested for validity as a chronic pain biomarker [53]. While most studies of neural pain biomarkers seek to predict the pain VAS, it will be important for future research to identify what power bands may support different dimensions of pain (i.e., somatosensory, affective, cognitive) by analyzing alternative metrics (e.g., McGill Pain Questionnaire (MPQ) or pain unpleasantness). There remains a large gap in characterizing such power-band based biomarkers of naturalistic, spontaneous chronic pain; an sEEG trial can fill this gap. Ultimately, by combining recording with simultaneous stimulation in these areas, an sEEG trial period could inform adaptive DBS algorithms (for a thorough discussion of brain-based pain biomarkers see [42,54]).

### 5.2. Biomarkers of Stimulation-Induced Pain Relief

Beyond biomarkers that predict the chronic pain state, it is critical to characterize neural signals resulting from the electrical stimulation of various sites across many parameters. There are at least three strategies for detecting biomarkers of stimulation-induced pain relief. First, by recording LFPs from multiple brain regions either during or immediately after pain-relieving stimulation (see Section 6, Practical Considerations), spectral power within the frequency bands of interest can be compared to validate putative pain biomarkers that were detected in the absence of stimulation. For example, if ACC theta power was positively correlated with a high reported pain state, therapeutic stimulation would be expected to decrease ACC theta power if this biomarker was causal. Such a biomarker could be incorporated into a closed-loop algorithm using a simple threshold; for example, one algorithm could initiate stimulation when ACC theta power increased beyond some prespecified threshold power value. Second, neural activity in certain frequencies recorded at the site of stimulation may help predict whether and when stimulation may be beneficial. In a notable study, mechanistic evidence supporting the VPL/M as a target for both recording and stimulation was provided by Huang et al. in thirteen patients with chronic pain [55]. Distinct thalamic theta, alpha, high beta, and high gamma oscillations were correlated with pain relief and, thereby, suggested possible biomarkers by which one may identify individual patients who may benefit from VPL/M stimulation. Third, simultaneous recordings across multiple electrodes could provide a readout of functional connectivity in the pain network, which could be “pinged” by delivering intermittent single-pulse electrical stimulation (SPES) at various sites. SPES has previously been used to probe cortico-cortical connections and intra-areal plasticity by averaging LFP time-locked to the stimuli using sEEG in epilepsy patients [56]. The averaged LFP reflects cortico-cortical evoked potentials that can be recorded in adjacent and remote sites during periods of high and low pain states to provide a connectome representation of network changes related to chronic pain. Similar biomarkers of stimulation-induced pain relief can help to narrow down final candidate brain targets for permanent DBS implantation.

## 6. Practical Considerations for Chronic Pain DBS Trial Period

There are numerous factors to consider in the experimental design of a trial period: the duration of a trial period, how many patients to enroll, and outcome metrics of interest. Indeed, prior studies of DBS and chronic pain may have failed to reach primary endpoints because of nonrandomized design, heterogeneous patient populations, subjective assessment of patient outcomes, lack of measuring “meaningful” changes in symptoms, inconsistencies in sites stimulated, and other factors (see review [7]). Practical considerations should not be left to last minute planning. Common understanding of goals and procedures is needed across clinical specialties including neurological surgery, neurology, and pain management. The research team must work closely with neurosurgeons to map the targeting of electrodes for implantation; the administration of pain medications must be coordinated across pain providers, nursing staff, and research team so testing can be timed appropriately; patient care assistants and monitoring staff must be appraised of patient needs that differ from standard inpatient care on their unit; and research staff not accustomed to inpatient care must be oriented to the environment. We have found that participating in nursing staff meetings prior to patient implant is an effective way to coordinate activities and communicate DBS trial period goals between research staff, clinical personnel, and candidate patients.

The overall duration of implant for each patient should carefully weigh experimental need, insertional effect, patient tolerance, and infection risk (discussed further above). Experimental need can be broken down into multiple categories: resting-state biomarker discovery, stimulation efficacy, and modulation of task performance (task-based biomarker discovery/stimulation effects on task performance). Further, the insertional effect, or stun effect, which has been well-studied in Parkinson’s disease patients undergoing trial periods for DBS, can often be described as a temporary improvement in disease symptoms in response to DBS lead placement [57]. The positive effect can often persist 6 months following surgical implantation and before the onset of stimulation. The insertional effect has been similarly described in chronic pain patients, in which some patients (43% of 21 patients) experience a substantial reduction in their pain immediately following lead implant [58]. While the cause and predisposing conditions of this insertional effect are not well understood, Hamani et al. found that a positive insertional effect was correlated with a successful stimulation period.

An emerging trend has been to use the inpatient DBS trial period to both test stimulation and record neural activity that may guide biomarker discovery toward developing closed-loop DBS algorithms. Resting-state biomarker discovery is best served by recording periods of brain activity during wakefulness in the absence of stimulation or tasks and across varying levels of reported pain metrics. Level of pain may be assessed using a collection of surveys or verbal reports that are repeatedly administered throughout the trial period. The dynamics of subjective pain experience varies widely across patients and pain subtypes [59]. As the timescale of pain predictability based on neural activity is relatively unknown, we opt to study brain activity for relatively long periods (e.g., 30 min) around each survey point to provide a starting point on studying individual dynamics/timescales. Each time a survey is administered translates to one data point; this should be repeated multiple times throughout the entire trial period. At least 10 data points, but preferably many more, are needed for a statistically valid association of neural activity with the symptom reports, necessitating a total trial duration of at least 3 days.

Evaluating for stimulation efficacy should involve testing at least two different stimulation locations and various parameters in order to identify putative therapeutic targets for permanent implant. Two critical factors should be considered during the design of stimulation efficacy testing: placebo effect and expected wash-in and wash-out durations. The placebo effect is an important confounder during DBS [60], and while it may be advantageous in the long-term treatment of chronic pain, care should be taken to control for short-term placebo effect during trial DBS. In order to account for this, double-blind, sham-controlled stimulation testing is needed. By design, when using clinically approved devices, the operator of the stimulator cannot be blinded to the stimulation condition. Thus, during double-blind stimulation testing, the individual operating the DBS stimulator should not interact with the patient in any way, and ideally should not be visible to the patient. All other people in the room (including the patient) should be blinded to the stimulation condition. Furthermore, it can be useful for all other people in the room and the patient to be naïve of stimulation (sham) onset and offset times. This may further mitigate expectation or anxiety associated with anticipated effects of stimulation, which may offer better predictive value of long-term success. Having a sham block paired with each verum stimulation block doubles testing time but provides more robust and interpretable results. If possible, having the patient self-administer surveys at cued times, with no researchers in the room, may also decrease Hawthorne effects [61]. This may not always be feasible due to safety considerations and the need for active monitoring of intracranial recordings during stimulation. The duration of stimulation and the wash-out period between testing sham/verum blocks or between stimulation locations should be determined as a function of expected wash-in and wash-out effects. While stimulation of some targets such as the VPL or PAG can produce rapid pain relief over seconds to minutes, the ACC or other sites may have much longer wash-in times over days. This remains an area of active investigation and varies as a function of disease duration and specific behavior/symptom assessed [16,62,63,64]. Longer wash-out periods provide more confidence that effects observed for subsequent stimulation parameters cannot be attributed to carryover effects from prior stimulation conditions. All candidate stimulation targets should be tested multiple times, so counterbalancing test order can also help mitigate carryover effects. Early in target discovery, it may be beneficial to stimulate different parameters within the same region (e.g., low vs. high pulse width or frequency) with shorter wash out; if a candidate brain region or parameter set for therapeutic efficacy is found, it is useful to retest it with longer wash out.

Recording neural activity during behavioral tasks, in the presence and absence of stimulation, can serve multiple purposes. Specifically, we propose using behavioral tasks that systematically assess various domains of the pain experience including somatic, affective, and cognitive ones. Tasks that are particularly relevant include quantitative sensory testing with thermal stimuli including pain thresholds [65] and mechanical pain thresholds with Von Frey filaments [66] (somatic); the emotional Stroop [67] and emotional facial recognition tasks (affective judgment and interference); and the oddball [68]; and Iowa gambling tasks [69] (cognitive attention and decision making). Task-based biomarkers can be used to track symptom status and treatment efficacy or identify brain regions that may be best targeted by stimulation for therapeutic benefit [70]. Machine learning methods can be applied to identify spectral properties that could serve as biomarkers for specific symptoms or induced states [71]. Variation in task performance may also more directly indicate the efficacy of stimulation. In order to make such conclusions, patients must be familiarized with tasks in order to prevent test–retest effects from skewing the results. Initial familiarization with tasks can be conducted prior to implant. Following implantation, tasks must be conducted multiple times (e.g., with and without stimulation) in order to determine baseline activity vs. potential changes induced by stimulation.

Not to be underestimated, patient tolerance and ability to actively participate during the trial period is critical for success. Detailed informed consent, ideally implementing teach-to-goal methods [72], should include clearly detailed information about what to expect during the trial period. In addition, we have found that having the patient complete questionnaire surveys (similar to those subsequently administered during the trial period) in his/her home environment prior to implant can decrease frustration with completing the same metric repeatedly. Continued clear communication with the patient and family members is vital. Having well-defined times when family members can visit or share a meal provides needed breaks in long days of testing. The depth of scientific discussion will need to be tailored to each patient’s interest and level of understanding, but overall, we have found that patients are much more engaged when they clearly understand the need for each study and the role each test plays in the larger picture.

The selection of which metrics should be administered to assess pain and how often is again tied to both scientific need and patient tolerability. Patients experience survey fatigue in answering the same surveys repeatedly (e.g., upward of 50 times a day), which can hinder accurate communication of symptoms. Individual patents also differ in their adeptness of communicating pain through numeric ratings, free speech dialog, pain maps [73], or standardized surveys. Different methods of collecting information about pain symptoms also tap into different aspects of the pain experience [74], such as quantitative pain measures (e.g., numerical rating), pain experience (e.g., unpleasant sensory or emotional experience), and pain expression (e.g., qualitative words, pain narrative, and behavior to communicate pain, pain behavior). We found that a combination of metrics was the most useful, with short versions administered more frequently and long versions administered about three times per day.

When deciding how many people to enroll in a trial period, overall expense, and expected trial to permanent conversion ratio must be taken into consideration. Expense (both in time and money) impose a cap on the total number of patients who can be enrolled. A meta-analysis review of spinal cord stimulation showed a 41% conversion ratio from a trial period to a permanent implant [4]. While a higher conversion ratio may seem preferable, imposing increasingly strict inclusion criteria on a trial period may prevent testing in a broader population who could ultimately benefit from the therapy. Regardless, given the intensiveness of the trial period and subsequent management of patients with implanted devices, the size of DBS trials for chronic pain will likely remain small (<30 individuals) for the foreseeable future, until the therapy is sufficiently developed to obtain regulatory approval.

Lastly, we must balance the goal of full mechanistic understanding of neural circuitry underlying chronic pain vs. the identification of effective therapy. It is not practical to require the former as a prerequisite for providing treatment options for patients currently suffering from chronic pain. Rather, we recommend incorporating our current knowledge of pain circuits and using this as a starting point for where to implant electrodes for biomarker discovery. This approach has the benefit of not only being ready for implementation now but also being mechanistically informed according to current state of knowledge. Existing therapies, including spinal cord stimulation, have been shown to be effective at alleviating chronic pain in many individuals, but the mechanisms remain debated. Most important for clinical treatment is the efficacy of the therapy. While the number of patients implanted remains modest, it is likely more feasible to focus on a more individualized approach. Recording from implicated regions affords the relatively rare opportunity to identify correlative or causal factors that predict pain or pain relief in individual subjects. However, as the cohort of patients grow, looking for trends across patients will be incredibly valuable to determine whether patients share common biomarker features. This may be particularly fruitful to separate cohorts with different pain etiologies, pain dynamics, or even based on syndrome subtypes.
